# Biocidal Activity of Tannic Acid-Prepared Silver Nanoparticles towards Pathogens Isolated from Patients with Exacerbations of Chronic Rhinosinusitis

**DOI:** 10.3390/ijms232315411

**Published:** 2022-12-06

**Authors:** Joanna Szaleniec, Agnieszka Gibała, Joanna Stalińska, Magdalena Oćwieja, Paulina Żeliszewska, Justyna Drukała, Maciej Szaleniec, Tomasz Gosiewski

**Affiliations:** 1 Department of Otolaryngology, Faculty of Medicine, Jagiellonian University Medical College, Jakubowskiego 2, 30-688 Krakow, Poland; 2 Jerzy Haber Institute of Catalysis and Surface Chemistry Polish Academy of Sciences, Niezapominajek 8, 30-239 Krakow, Poland; 3 Department of Molecular Medical Microbiology, Faculty of Medicine, Jagiellonian University Medical College, Czysta 18, 31-121 Krakow, Poland; 4 Department of Cell Biology, Faculty of Biochemistry, Biophysics and Biotechnology, Jagiellonian University, Gronostajowa 7, 30-387 Krakow, Poland

**Keywords:** chronic rhinosinusitis, exacerbations, microbiome, microbiota, bacteria, antibiotic resistance, silver nanoparticles, tannic acid

## Abstract

The microbiome’s significance in chronic rhinosinusitis (CRS) is unclear. Antimicrobials are recommended in acute exacerbations of the disease (AECRS). Increasing rates of antibiotic resistance have stimulated research on alternative therapeutic options, including silver nanoparticles (AgNPs). However, there are concerns regarding the safety of silver administration. The aim of this study was to assess the biological activity of tannic acid-prepared AgNPs (TA-AgNPs) towards sinonasal pathogens and nasal epithelial cells (HNEpC). The minimal inhibitory concentration (MIC) for pathogens isolated from patients with AECRS was approximated using the well diffusion method. The cytotoxicity of TA-AgNPswas evaluated using an MTT assay and trypan blue exclusion. A total of 48 clinical isolates and 4 reference strains were included in the study (*Staphylococcus aureus*, *Pseudomonas aeruginosa*, *Escherichia coli*, *Klebsiella pneumoniae*, *Klebsiellaoxytoca*, *Acinetobacter baumannii*, *Serratia marcescens*, *Enterobacter cloacae*). The results of the studies revealed that the MIC values differed between isolates, even within the same species. All the isolates were sensitive to TA-AgNPs in concentrations non-toxic to human cells during 24 h exposition. However, 48 h exposure to TA-AgNPs increased toxicity to HNEpC, narrowing their therapeutic window and enabling 19% of pathogens to resist the TA-AgNPs’ biocidal action. It was concluded that TA-AgNPs are non-toxic for the investigated eukaryotic cells after short-term exposure and effective against most pathogens isolated from patients with AECRS, but sensitivity testing may be necessary before application.

## 1. Introduction

Chronic rhinosinusitis (CRS) is an inflammatory disease of the sinonasal mucosa. An understanding of the complex relationships between the inflammatory process and the microbiota of the sinuses is still in its infancy. The role of bacteria seems to be more evident in acute exacerbations of chronic rhinosinusitis (AECRS). If the sudden worsening of symptoms is accompanied by purulence in the sinuses, the incident is attributed to bacterial infection and antibiotic treatment is recommended [1,2]. Increasing antibiotic resistance of sinonasal pathogens is one of the factors that has limited treatment efficacy in AECRS [3]. As a result, there is growing interest in novel therapeutic options.

Intranasal preparations of silver nanoparticles (AgNPs) have been proposed as an alternative treatment for sinonasal infections if antibiotic therapy is ineffective. AgNPs are objects of any shape and dimensions in the range from 10^−9^ m to 10^−7^ m built from silver atoms. Depending on the methods and stabilizing agents used to synthesize the AgNPs, they differ significantly in their physicochemical and biological properties [4,5]. The AgNPs dispersed in liquid media are sometimes referred to as “colloidal silver” [6]. However, this term is not precise and may lead to misunderstandings. Many over-the-counter preparations marketed as “colloidal silver” contain various unspecified forms of silver but no well-characterized silver nanoparticles [7]. 

Potential medical applications of AgNPs have evoked either enthusiasm or serious concerns. The antimicrobial properties of silver have been known for millennia. Silver has been shown to be effective against a wide variety of microbes, including Gram-positive and Gram-negative bacteria, yeast and fungi [8]. It damages many vital structures in the cells and therefore, it was believed that resistance to silver was very unlikely to emerge [9,10]. Unfortunately, recent studies have proven that bacteria can develop manifold mechanisms of silver resistance, some of which additionally result in cross-resistance to antibiotics [11]. Research on AgNP toxicity has provided contradictory results. Some studies have reported the acceptable safety of AgNP preparations [6,12,13], while others have provided alarming data on cytotoxicity and accumulation in many organs, including the brain after intranasal delivery [14]. The toxicity of AgNPs undoubtedly depends on dosing and formulation, but reliable data on the optimal method of administration in sinonasal infections have not been established. 

Recent studies have shown that bacteria in clinical settings sometimes harbor silver resistance genes, and occasionally, the clinical isolates can tolerate exceedingly high silver concentrations [15]. These findings undermine the conviction that silver is a universal antimicrobial and can be used for any infection without previous sensitivity testing. However, due to the lack of routine screening, the incidence of silver resistance in human infections is unknown. 

On the other hand, numerous reports in the literature have shown that the biocidal activity of AgNPs strongly depends on their surface properties, such as surface charge and the chemistry of stabilizing layers. The surface properties of AgNPs can be shaped using selected stabilizing agents. It seems plausible that AgNPs coated by natural compounds exhibiting desired biological activity should be most beneficial for medical applications. Recently, the attention of scientists was paid to the stabilization and utility of AgNPs capped by diverse polyphenol molecules. For instance, Galdopórpora et al. [16] showed that core-shell-coated polyphenol AgNPs possess antimicrobial and antioxidant activities. Moreover, it was established that polyphenol-capped AgNPs are biocompatible in low concentrations. In turn, Rezazadeh et al. [17] found that synergistic antibacterial effects appear when AgNPs are combined with chitosan and polyphenol biomolecules. 

It seems that tannic acid (TA) is one of the most popular and widely used middle molecular mass polyphenols involved in the preparation of AgNPs. At the level of AgNP synthesis, TA plays a dual role because it reduces silver ions and stabilizes formed nanoparticles. From a biological point of view, TA is a well-known antibacterial and antiviral agent [18]. The efficiency of TA was confirmed towards such viruses as human immunodeficiency virus (HIV), herpes simplex virus (type 1 and 2) and Noroviruses, to name a few [18,19,20]. It is worth mentioning that recently it was found that TA is also a promising candidate for preventing and inhibiting the infectivity of SARS-CoV-2 [21]. In turn, the antibacterial activity of TA has been proven on Gram-positive and Gram-negative bacteria, including other *Staphylococcus aureus, Escherichia coli*, *Yersinia enterocolitica* and *Listeria innocua* [18].

Numerous scientific articles have shown that TA is a promising candidate in the deactivation of diverse types of cancer cells. Mhlanga et al. [22], investigating the impact of TA on human liver hepatocellular carcinoma (HepG2) found that this polyphenol induces cell apoptosis by DNA fragmentation via caspase-dependent and independent mechanisms. Moreover, TA induces oxidative stress in the cells leading to their death [22]. Similar observations were noticed by Sp et al. [23], who studied the apoptosis mechanism in human embryonic carcinoma cells (E.C., NCCIT). It was established that TA is able to induce extrinsic apoptosis in NCCIT cells by regulating mitochondrial reactive oxygen species (mROS). Overall, a potential anticancer activity of TA against several solid malignancies such as liver, breast, lung, pancreatic, colorectal and ovarian cancers has been reported [24].

Taking the aforementioned issues into consideration, our attention focused on the synthesis of AgNPs with the use of TA and evaluating their potential in treating sinonasal bacterial infections in patients with chronic rhinosinusitis. The main objective of our study was to assess if TA-AgNPs could be effectively applied to fight pathogens isolated from patients with AECRS. We tested the susceptibility to TA-AgNPs in 48 pathogens isolated from 50 patients with AECRS. To our knowledge, to date, this is the most extensive survey on AgNP susceptibility in CRS patients [6]. In parallel experiments, we evaluated the toxicity of TA-AgNPs for nasal epithelial cells in vitro. Our purpose was to determine if there is a potential therapeutic window, i.e., a concentration of TA-AgNPs that is already effective against the pathogens but still safe for the host’s epithelium. The secondary goal was to evaluate the incidence of bacterial isolates that were resistant to TA-AgNPs at concentrations non-toxic for human cells.

## 2. Results

### 2.1. Synthesis and Physicochemical Characteristics of TA-AgNPs

**Figure 1:**
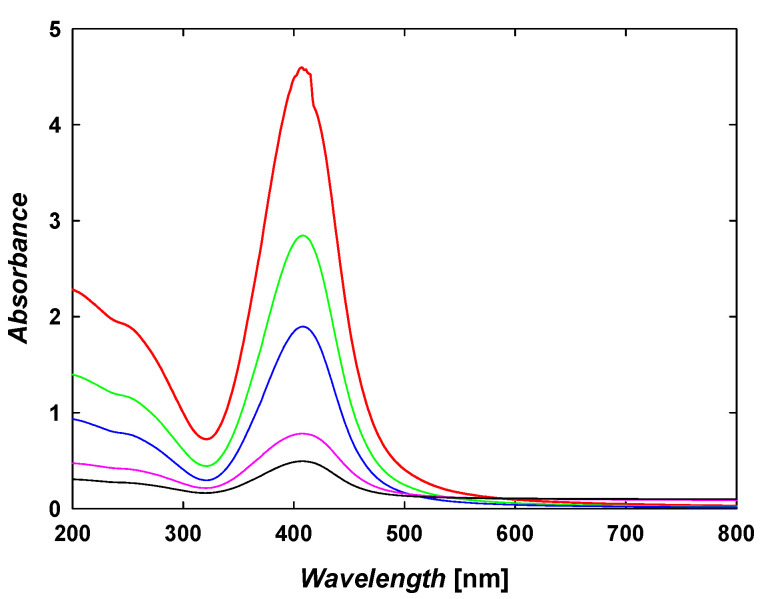
Extinction spectra of TA-AgNP suspensions of the controlled mass concentration of nanoparticles. Extinction spectra of TA-AgNP suspensions of the controlled mass concentration of nanoparticles (^___^) 5 mg L^-1^, (^___^) 10 mg L^-1^, (^___^) 20 mg L^-1^, (^___^) 25 mg L^-1^, (^___^) 50 mg L^-1^.

TA-AgNPs were obtained via a chemical reduction method of silver ions, delivered in the form of silver nitrate, by TA under alkaline conditions [5,25]. In contrast to other standard preparation protocols [26], no additional stabilizing agents were applied. An aqueous suspension of TA-AgNPs was purified from unreacted compounds using the ultrafiltration method described previously in detail [25]. The mass concentration of TA-AgNPs in the stock suspension was determined using inductively coupled plasma optical emission spectrometry (ICP-OES) and based on the results obtained from density measurements [25]. It was found that the mass concentration of TA-AgNPs in the stock suspension was equal to 217 ± 3 mg L^−1^. After the purification procedure, silver ions were not detected in the stock suspension. The pH of the suspension attained was equal to 5.8. The suspension exhibited an intense yellow color. Recorded extinction spectra showed that the maximum absorption band appeared at the wavelength of 412 nm (Figure 1). A typical increase in absorbance value with an increase in TA-AgNP mass concentration in the aqueous suspension was observed at the wavelength of 412 nm. This finding remains in agreement with other literature reports showing an absorption increase with silver content in the colloidal suspension [27,28].

**Figure 2:**
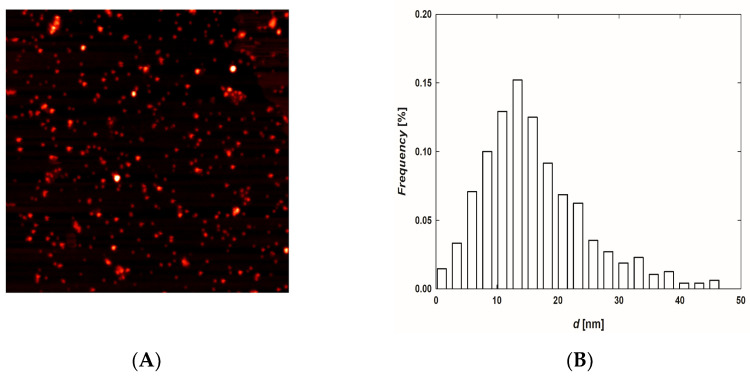
(**A**) Typical AFM image (2 μm × 2 μm) presenting TA-AgNPs deposited on PAH-modified mica and (**B**) size distribution of nanoparticles.

TA-AgNPs were deposited on poly (allylamine hydrochloride) (PAH)-mica sheets [29] and were imaged using atomic force microscopy (AFM). A typical AFM image of deposited TA-AgNPs and the size distribution of nanoparticles are presented in Figure 2. It was established that TA-AgNPs exhibited a quasi-spherical shape and an average size of 16 ± 4 nm. The results obtained based on AFM imaging remained in agreement with the findings from micrographs recorded using transmission electron microscopy (TEM) [5]. 

TA-AgNPs dispersed in the suspension were also characterized by the use of dynamic light scattering (DLS) and electrophoretic light scattering (ELS) [5]. The hydrodynamic diameter of TA-AgNPs was 12 ± 5 nm. It is worth mentioning that this value is consistent with the hydrodynamic diameter of AgNPs described previously [25]. This confirms that the preparation procedure of TA-AgNPs is highly reproducible. The electrokinetic measurements showed that the electrophoretic mobility of TA-AgNPs, determined using the stock suspension at the temperature of 20 and 37 °C, reached a value of −2.96 ± 0.23 and −3.30 ± 0.23 μm cm (Vs)^−1^, respectively. The zeta potential of TA-AgNPs was calculated based on Henry’s model [29]. TA-AgNPs were negatively charged at pH 5.8 and characterized by the zeta potential equal to −52 ± 3 mV at the temperature of 37 °C. Previously, the zeta potential equal to −58 ± 2 mV was reported for AgNPs prepared with the use of TA by Orłowski et al. [19]. This evidence indicates that the control of the electrokinetic properties of TA-AgNPs was also maintained for the systems prepared in different laboratories. 

### 2.2. Bacterial Isolates

**Figure 3:**
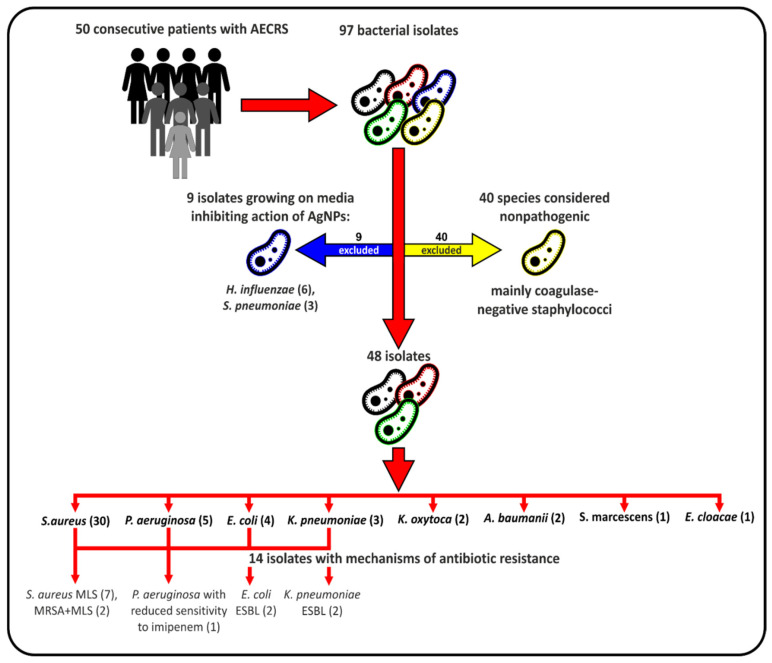
Flow chart detailing the election of bacterial isolates for the study.

Swabs from the sinonasal cavity were obtained from 50 patients with acute exacerbations of chronic rhinosinusitis (AECRS). The group included 27 women and 23 men aged 25–80 (mean age 51). All of the patients had undergone endoscopic sinus surgery in the past, so their sinuses were accessible for sampling. Most of the participants had chronic rhinosinusitis with nasal polyps (90%) and presented with comorbidities such as asthma (54%), aspirin-exacerbated respiratory disease (10%) or allergy (38%). As presented in the flow chart in Figure 3, the sensitivity to TA-AgNPs was tested in 48 out of 97 isolates. Species considered nonpathogenic and bacteria that required media that inhibit the TA-AgNPs were excluded.

### 2.3. Determination of the Minimal Inhibitory Concentration (MIC)

**Figure 4:**
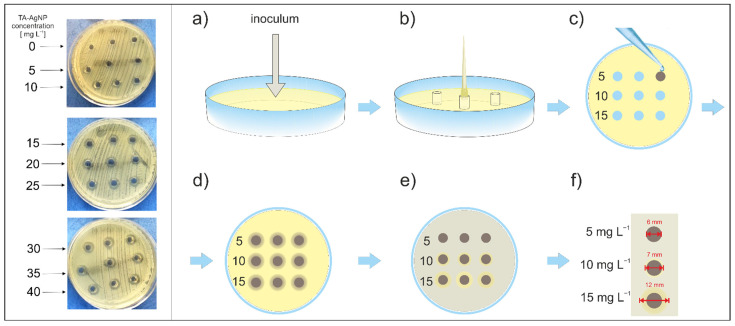
The well diffusion method of MIC approximation used in the study; Left panel: photograph of experiment with *E. coli* isolate and TA-AgNP in the concentration range of 0–40 mg L^−1^ (MIC upper limit: 15 mg L^−1^) Right panel: schematic representation of the experimental protocol: (**a**) inoculation with isolate saline suspension 10^6^ CFU mL^−1^; (**b**) wells in Müller–Hintonagar were cut with a sterile pipette tip; (**c**) 100 μL TA-AgNP suspension was added into each well in a concentration range of 5–100 mg L^−1^, in the figure triplicates of 5, 10 and 15 mg L^−1^ (**d**) diffusion of TA-AgNP in the Müller–Hinton agar during 4 h incubation at 4 °C; low temperature slowed down the bacteria growth, (**e**) bacteria growth over Müller–Hinton agar during 18 h incubation at 37 °C; (**f**) measurement of a zone of inhibition of bacterial growth and estimation of MIC (here MIC upper limit 10 mg L^−1^) Colors used in the figure: yellow—Müller–Hinton agar, brown—TA-AgNPs suspension added to the wells (**c**) and diffusing in the agar (**e**), grey—bacterial growth.

Evaluation of MIC in a liquid medium using turbidimetric measurements turned out to be impossible due to interference between TA-AgNPs at higher concentrations and the instrument’s readouts. To overcome this problem, an alternate, well diffusion method [30,31] was applied, which approximated MIC according to the procedure presented in Figure 4 and described in detail in the Materials and Methods Section.

The upper limit of minimal inhibitory concentration (MIC) values of TA-AgNPs for the clinical isolates and reference strains are presented in Table 1. Higher MIC values indicated a lower sensitivity to TA-AgNPs. Every experiment was performed in triplicate. The method of MIC determination was semiquantitative and provided results with an accuracy of up to 5 mg L^−1^. The MIC values determined by this method were highly reproducible. The same result was noted for all three replications (SD = 0) for every isolate. The MIC values for the clinical isolates ranged from 5 to 40 mg L^−1,^ with a median value of 10 mg L^−1^. For each of the identified species, the sensitivity to TA-AgNPs varied between isolates.

The lowest median MIC value was noted for *P. aeruginosa* followed by *S. aureus* and *A. baumannii* while the highest values were observed for the other Gram-negative rods. However, the differences in sensitivity between species were not statistically significant due to high variability within each species. There were no significant differences between Gram-negative bacteria and Gram-positive bacteria (in this study represented only by *S. aureus*). It is also apparent that the MIC values for reference strains cannot be considered representative of the clinical isolates. The reference strain of *P. aeruginosa* ATCC 27857 used in the study was less sensitive to TA-AgNPs than *S. aureus* ATCC 29213, *E. coli* ATCC 25922 or *K. pneumonia* ATCC 31488, while the results obtained for clinical isolates showed a reversed trend.

Antibiotic resistance mechanisms were identified in 14 clinical isolates. Resistance associated with extended-spectrum beta-lactamases (ESBL) was identified in two *E. coli* isolates, two *K. pneumoniae* isolates and one *A. baumannii* isolate. Among the *S. aureus* isolates, ninewere resistant to macrolides, lincosamides and streptogramins (MLS). Two of these isolates were additionally resistant to methicillin (MRSA). Median MIC values for the antibiotic-resistant isolates were higher than for antibiotic-sensitive bacteria, but this difference was not statistically significant (Table 2). However, the number of antibiotic-resistant bacteria in the study was probably too low to observe possible more subtle relationships.

Finally, as our AgNPs were functionalized with TA, which has been reported to exhibit bacteriostatic or bactericidal effects [18,32,33], we investigated TA’s impact on the growth of two representative strains of *S. aureus* (ATCC 29213) and *E. coli* (ATCC 25922) using the protocol used to estimate MIC for TA-AgNPs. Noteworthy, the TA’s final concertation on the surface of the AgNP is unknown. As a result, we investigated its effectiveness over a wide concertation range, starting from that used to synthesize the AgNP stock suspension (55 µM) down to a 100-time diluted solution. Only when a non-diluted TA was applied to the wells we observed a zone of inhibition for both control strains. Such concertation would potentially be available only if we used undiluted TA-AgNPs, that is, TA-AgNPs at a concertation of 214 mg L^−1^. As the maximum upper limit of MIC reported in that study was 40 mg L^−1^ we assumed that any independent effect of TA on bacteria was probably below the detection level of the well diffusion method.

### 2.4. Cytotoxicity of TA-AgNPs towards Human Primary Nasal Epithelial Cells (HNEpC)

**Figure 5:**
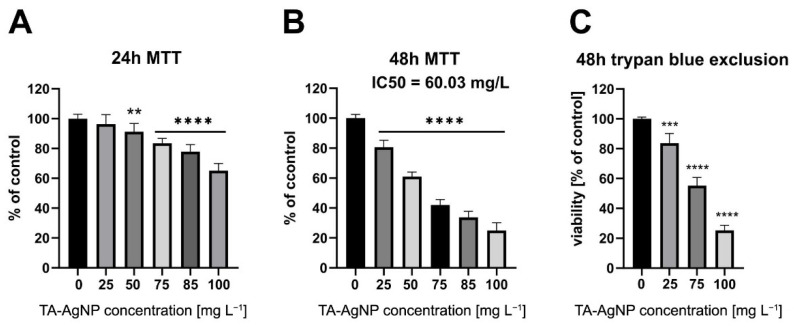
Cell viability of human primary nasal epithelial cells. (**A**) MTT assay following exposure of cells to TA-AgNPs at the indicated concentrations for 24 h. (**B**) MTT assay following exposure of cells to TA-AgNPs at the indicated concentrations for 48 h. Data represent mean values of three independent experiments performed in triplicate (*n* = 9), expressed as % of control ± S.D. * indicates values significantly different from the control, i.e., MTT assay for 0 mg L^−1^ of TA-AgNP. (**C**) Trypan blue exclusion assay. The percentage of viable cells was determined after a 48 h incubation in the presence of TA-AgNPs. Data represent mean values ± S.D. Statistical significance compared to the control assays at 0 mg L^−1^ of TA-AgNP. Levels of statistical significance: ** *p* ≤ 0.01; *** *p* ≤ 0.001; **** *p* ≤ 0.0001.

**Figure 6:**
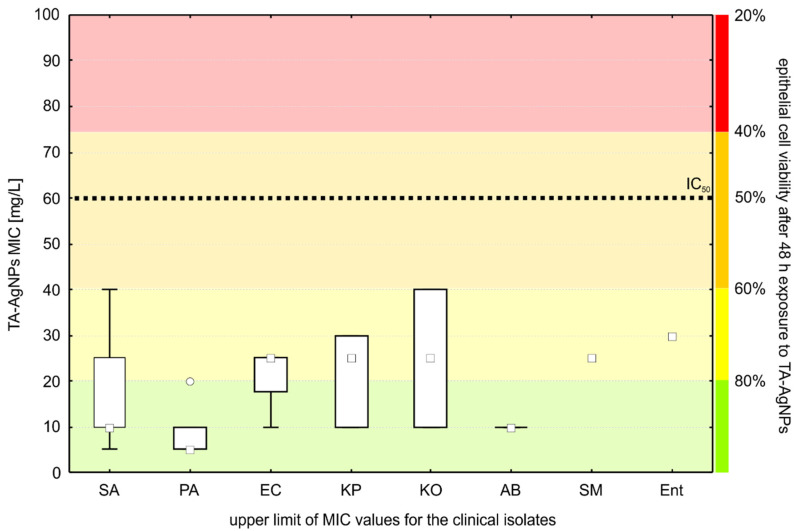
MIC upper limits for the clinical isolates compared to the nasal epithelial cell viability after 48 h exposure to TA-AgNPs (IC_50_—the half-maximal inhibitory concentration). SA—*Staphylococcus aureus*, PA—*Pseudomonas aeruginosa*, EC—*Escherichia coli*, KP—*Klebsiella pneumoniae*, KO—*Klebsiella oxytoca*, AB—*Acinetobacter baumannii*, SM—*Serratia marcescens*, Ent—*Enterobacter cloacae).* The boxes represent the interquartile range; the whiskers extend to the highest and lowest values, the squares represent the median values, and the circle represents an outlier.

The cytotoxicity of TA-AgNPs was assessed in the in vitro culture of human primary nasal epithelial cells (HNEpC). The results of the studies are presented in Figure 5. Incubation in the presence of TA-AgNPs reduced the viability of epithelial cells in a dose-dependent manner. However, high concentrations of TA-AgNPs and prolonged incubation time were required to achieve cytotoxic effects. At 24 h of incubation, the cell viability remained above 80% for up to 75 mg L^−1^ TA-AgNPs, and no morphological changes implicating cell death were observed. At the highest concentration tested (100 mg L^−1^), the cell viability measured with the MTT assay was 65.11% ± 4.86%, suggesting that human nasal epithelial cells were much less sensitive to TA-AgNPs than any of the tested bacterial isolates. The half-maximal inhibitory concentration (IC50) for the 48 h time point was determined at 60.03 mg L^−1^, which was also above the MIC for 100% of the tested pathogens. Viability of 80% after 48 h was maintained at 25 mg L^−1^. This concentration of TA-AgNPs was effective against 39 (81%) of the isolates (Figure 6).

The findings of the colourimetric assay were confirmed with a cell death assay based on cell membrane integrity (trypan blue exclusion assay). Results obtained using both methods were comparable, which indicates that the lower absorbance values observed in the MTT assay were due to the changes in cell viability and not to changes in cell mitochondrial activity or cytostatic effects of the nanoparticles.

## 3. Discussion

In this study, we examined the sensitivity of sinonasal pathogens to AgNPs obtained with the use of tannic acid (TA). TA-AgNPs were obtained in the form of the aqueous suspension according to the well-established preparation protocols [5,20,25]. The physicochemical characteristics of TA-AgNPs revealed that they are characterized by a quasi-spherical shape and a negative zeta potential. Comparing the experimental data with the literature reports [19,20], we concluded that the preparation of AgNPs with the use of TA is reproducible. It was established that by applying TA, one could obtain AgNPs of controlled properties.

Having well-defined TA-AgNPs, the attention was focused on the evaluation of their bactericidal properties. The well diffusion method [30,31] was applied to approximate the minimal inhibitory concentration (MIC), as shown in Figure 4. The MIC values of the clinical isolates ranged between 5 and 40 mg L^−1^. The values reported by other authors for the same bacterial species vary between studies due to differences in the type of AgNPs, bacterial strains and methods used for sensitivity testing [8,34,35,36,37]. Gangwar et al. [38] studied the antimicrobial activity of tannic acid-prepared AgNPs that were similar to those used in our study. They reported much higher values of MIC (64 mg L^−1^ for a reference strain of *P. aeruginosa*, >64 mg L^−1^ for reference strains of *S. aureus*, *E. coli*, *K. pneumoniae* and *A. baumannii*). However, they used the disk diffusion method, where paper discs were dipped in the TA-AgNPs solution and subsequently placed on the surface of agar inoculated with bacteria. It has been shown that this approach is less sensitive than the well diffusion method used in our study [39]. The diffusion rate of AgNPs from the paper disks to the agar is unknown. Therefore, the actual concentration of TA-AgNPs in the medium surrounding the disc may be much lower than the initial concentration of the TA-AgNPs solution. In the well diffusion method, the concentration of nanoparticles around the well is most probably higher than the concentration around the paper disk. We are aware that it may still be lower than the initial concentration of the stock suspension. Therefore, the value of ‘MIC’ established in our study should be interpreted as the upper limit of actual MIC (which means that the actual MIC is not higher than the lowest concentration in the well with a zone of inhibition). Nevertheless, the well diffusion method likely provides more accurate approximations of MIC than the disk diffusion method.

Furthermore, using only one reference strain to represent a species is a common practice. Our research proved that this approach is incorrect. We showed that the sensitivity to AgNPs in reference strains was not representative of the values noted for clinical isolates of the same species. The upper limit MIC values for the reference strains of *S. aureus*, *E. coli* and *K. pneumoniae* were two to five times lower than the median values for the clinical isolates. In contrast, the reference *P. aeruginosa* was characterized by the upper limit of MIC values that were twice as high as the median value for clinical isolates of the same species. Among the clinical isolates, there were considerable differences in the upper limit of MIC values between isolates of the same species. These differences were not species-specific. For most species, we found isolates that were highly sensitive to TA-AgNPs (MIC < 5 mg L^−1^) as well as isolates that were inhibited only by much higher concentrations of the antimicrobial (40 mg L^−1^).

We also laboured to assess the potential antimicrobial effect of TA component of TA-AgNPs. The tests conducted on two reference strains (*E. coli* and *S. aureus*) confirmed the inhibitory effect of TA on bacterial growth. However, the observable effect was noticeable only at high concentrations of TA (55 µM, i.e., 94 mg L^−1^), beyond which bacteria were subjected to in the experiments with TA-AgNPs. Such a high value of MIC is consistent with results previously reported by Tae Yoon Kim et al. [40], who reported MIC of TA for several reference strains in the range of 53–425 mg L^−1^. Our previous experiments suggested synergistic effects of TA and AgNPs: TA-AgNPs had a higher antibacterial activity compared to other AgNPs functionalized with different agents. [5]. Still, the functionalizing agent also influences the size, charge and stability of AgNPs; therefore, conclusions regarding potential synergy should be approached with caution.

Differences in the cell wall structure between Gram-positive and Gram-negative bacteria have been shown to influence their interactions with metal nanoparticles [10]. In several studies, the Gram-negative *E. coli* was more susceptible to AgNPs than the Gram-positive *S. aureus* [41], with MIC values 2–10 times lower depending on the study [34,35]. On the contrary, in our study, there were no statistically significant differences in MIC values between Gram-positive and Gram-negative bacteria, but the mean MIC value for Gram-positive isolates was lower than for the Gram-negative isolates. TA-AgNPs used in our study as well as described by other researchers [19,38,42,43] were characterized by negative zeta potential. Therefore, it can be assumed that repulsive electrostatic interactions occur between the AgNPs and the LPS in the cell wall of the Gram-negative bacteria [10]. However, the experimental results showed that these electrostatic interactions do not play a crucial role in the AgNP bactericidal activity. Numerous reports in the literature have indicated that TA-synthesized AgNPs are highly efficient in the deactivation of bacteria as well as the reduction of microbial biofilms [44].

Previously, the efficacy of AgNPs against clinical isolates from patients with CRS and otitis was assessed by Feizi et al. [6]. The AgNPs were prepared using *Corymbia maculate* extract. Eighteen bacterial strains were included in the study (five MRSA, five *P. aeruginosa*, five *H. influenzae*, three *S. pneumoniae*). *P. aeruginosa* was more sensitive to the AgNPs than other species, but in general Gram-positive isolates were not universally more resistant than Gram-negative bacteria. The authors also noted that MIC values differed among the strains for each species. The study was conducted in Australia and utilized different AgNPs than the ones used in our research, but the conclusions were strikingly similar to our observations carried out in Europe. Both studies showed that the sensitivity of sinonasal pathogens to AgNPs is not easily predictable. If AgNPs are used in therapy, each isolate obtained from a patient needs to be tested individually to determine possible resistance.

Recent observations suggest that the uncontrolled application of silver without prior knowledge about the pathogen’s sensitivity may result in exposition to sublethal concentrations and promote resistance development. Bacteria can develop multiple resistance mechanisms that protect them from silver and AgNPs [9,10]. It was shown that resistance could be rapidly induced after exposure to increasing concentrations of silver [11], suggesting that silver preparations should be applied with caution. Just like antibiotics, they need to be used only when antimicrobial treatment is clearly indicated, and the dosage must be adequate to achieve concentrations above the MIC values of the targeted pathogen.

Resistance to silver was also shown to promote cross-resistance to antibiotics [11,45,46]. In our study, 37.5% of the isolates had antibiotic resistance mechanisms. We observed higher MIC values for these isolates compared to antibiotic-sensitive bacteria, but the differences did not reach statistical significance.

Studies reporting the prevalence of silver resistance in clinical settings are scarce. However, their results to date seem to be reasonably optimistic. Most data on the subject are related to bacteria isolated from burns, ulcers and other wounds frequently treated with silver dressings. Although silver resistance genes were encountered in 2–6% of clinical isolates, genetic resistance usually did not translate into phenotypic resistance [15,47,48,49]. Nevertheless, the first strains that could tolerate silver concentrations up to 5500 μM and were resistant to many commercially available wound dressings were occasionally found in environments where exposition to silver is more frequent [15,50,51]. Bacteria that do not display overt Ag-resistance frequently harbour genes that cause cryptic resistance, which is readily activated upon silver challenge [52]. These results indicate that silver resistance, although currently rare, may become an increasing problem in the future in case of unlimited overuse of silver preparations.

The question of whether AgNPs can be safely used as antimicrobial drugs in humans is under debate. Silver preparations are available over-the-counter despite a lack of adequate regulatory approval [7]. The toxicity levels reported for eukaryotic cells vary and depend on the properties of the AgNPs used in the experiments and the type of cells under consideration (15 mg L^−1^ for alveolar epithelial cells, 30 mg L^−1^ for monocytes, 80 mg L^−1^ for HeLa epithelial cells) [53,54,55]. Gangwar et al. [38] reported results on the cytotoxicity of TA-AgNPs to human lung carcinoma cells at approximately 32 mg L^−1^ (300 µM). Unfortunately, the study did not report cytotoxicity for non-cancerous control cells, which would allow a better comparison with our experiments.

The toxicity of AgNPs for human cells was compared with the toxicity of sinonasal pathogens in two studies. Feizi et al. [6] studied 18 clinical isolates from patients with CRS and otitis media. As it was mentioned above, the AgNPs for this study were produced using *Corymbia maculate* leaf extracts. The AgNPs were not toxic to human immortalized bronchial epithelial cells Nuli-1in concentrations equal to MIC for the sinonasal pathogens and up to 175 ppm after 1 h of exposure [6]. Chen et al. [56] studied the antibacterial properties of commercially available 10 nm AgNPs that were effective against reference strains of *E. coli* and *S. aureus* at a concentration of 5 ppm. They found that human nasal squamous cell carcinoma cells (RPMI2650) maintained >80% viability after 24 h of exposure to 5 ppm of AgNPs [56].

In our study, to assess the cytotoxicity of TA-AgNPs, we used primary HNEpC cells, which are the most reliable two-dimensional in vitro model of the nasal epithelium [57]. We observed that TA-AgNPs were non-toxic to epithelial cells in concentrations that inhibited the growth of all bacterial isolates tested when incubated with the cells for 24 h. At 48 h of incubation, TA-AgNPs were not cytotoxic up to 25 mg L^−1^, which is above the median MIC value for the tested bacteria. However, nine (19%) of the clinical isolates were not sensitive to TA-AgNPs at this concentration.

It is debatable what time of exposure in cytotoxicity experiments is representative of the real-life pharmacokinetics of the AgNPs in the nose and the sinuses. In the study conducted by Feizi et al. [6], the incubation time was very short (1 h). The authors explained that the mucociliary clearance eliminates the AgNPs within about 15 min when applied in a nasal solution [6]. However, to fight an infection, it will probably be necessary to maintain the drug concentration above the MIC value for a longer period. For antibiotics, the recommended treatment time for sinonasal infections is between 5 days and 3 weeks [1]. Therefore, it is reasonable to assume that AgNPs need to be used in a formulation that would increase the retention time of the nanoparticles in the nasal cavity or a dressing that would stay in contact with the nasal mucosa for prolonged periods.

We showed that TA-AgNPs were not toxic to HNEpC cells at concentrations above MIC for the sinonasal pathogens in the first 24 h of the experiments, but the cytotoxicity increased when the exposure time was longer. This observation suggests that the real-life toxicity of AgNPs may be higher than assumed from short-time experiments on cell lines. On the other hand, several studies have indicated that standard two-dimensional in vitro cell culture models may overestimate the cytotoxicity of different agents, including AgNPs [58,59]. Differentiated 3D in vitro models might more closely resemble what occurs in vivo due to barrier properties that reduce absorption across the stratified epithelium. Zavala et al. [59] showed that higher concentrations and longer exposure times of air pollutants are needed to induce cytotoxic effects in a 3D in vitro airway epithelium model compared to the 2D epithelial cell culture. Chen [56] found no toxicity of AgNPs in a 3D model of a stratified epidermis while observing a pronounced cytotoxic effect of equivalent doses of AgNPs in a 2D culture of keratinocytes. Similar 3D models of differentiated nasal epithelium cultured at the air–liquid interface, containing cilia and mucus-producing cells, have been described [60,61]. 

Local toxicity for nasal epithelial cells is not the only factor that limits the potential topical application of AgNPs in patients with CRS. Systemic distribution of AgNPs was observed after intranasal administration in rodent models. Aggregation of AgNPs was noted in the spleen, lung, kidney and nasal airway [14]. This resulted in enhanced destruction of erythrocytes in the spleen, but no other microscopic changes were associated with the AgNPs depositions. It has to be noted that the doses of AgNPs used in the study were exceedingly high (100–500 mg/kg). However, repeated inhalation of AgNPs for 28 days at concentrations of 10^6^ particles/cm^3^ caused no significant changes in rats’ nasal cavity or lungs, only an increase in the number and size of goblet cells. Moreover, direct nose-to-brain transport of AgNPs is suspected of bypassing the blood–brain barrier [14,62,63], but not all studies support this observation [7]. Importantly, the application of AgNPs results in significantly lower concentrations of silver in blood than the delivery of AgNO_3_ [7]. 

The safety of AgNPs in nasal rinses (15 mg L^−1^) in 11 patients with CRS was assessed by Ooi et al. [12]. Four patients had elevated serum silver levels, but no adverse events were reported and the silver levels did not reach the threshold for argyria. In our study, only 27 (56%) pathogens were sensitive to TA-AgNPs at concentrations of 15 mg L^−1^. However, Ooi et al. [12] applied negatively charged citrate-stabilized AgNPs and exhibited different physicochemical properties than TA-AgNPs, excluding direct comparisons.

Finally, we should mention the study limitations:

(a) The study group included only patients with postoperative AECRS, so the results presented in this paper may not apply to bacterial exacerbations of CRS in patients who did not undergo sinus surgery;
(b) Only aerobic species were included in the study;
(c) In this study, we used one type of AgNPs (TA-AgNP). The properties of AgNPs obtained with different methods may vary;
(d) This observational study was designed to estimate the prevalence of phenotypic resistance while the prevalence of resistance genes or cryptic resistance and determination of resistance mechanisms requires a more detailed exploration to explain the observed differences in sensitivity between bacterial isolates;
(e) The results of in vitro tests of AgNPs efficacy are always affected by the media in which the experiment is conducted due to their interaction with ions, macromolecules and blood [10]. Therefore, further research on the interactions between the AgNPs and the secretions in the sinonasal cavities is necessary before their application in vivo.

## 4. Materials and Methods

### 4.1. Sample Collection

The samples for this cross-sectional observational study were collected in the outpatient clinic of the Department of Otolaryngology of the University Hospital in Krakow in 2018 and 2019 (Jagiellonian University Medical College Bioethics Committee approval no. 1072.6120.208.2017). The eligibility criteria included: diagnosis of CRS according to EPOS 2012 diagnostic criteria [64], prior endoscopic sinus surgery (ESS), signs and symptoms of bacterial exacerbation [65] (worsening of symptoms, purulence in the sinonasal cavity) and no antibiotic therapy for at least a week. Swabs were collected under endoscopic guidance from the pathological secretions from the middle nasal meatus or the infected sinuses. Bacterial isolates were stored at −80 °C and thawed for experiments. Reference strains were obtained from the American Type Culture Collection (*Escherichia coli* ATCC 25922, *Staphylococcus aureus* ATCC 29213, *Pseudomonas aeruginosa* ATCC 27857, *Klebsiella pneumoniae* ATCC 31488).

### 4.2. Bacterial Identification, Antibiotic Sensitivity Testing and Identification of Antibiotic Resistance Mechanisms

The bacteria were inoculated on the Columbia blood agar with (OXOID) for Gram-positive aerobic cocci, on the chocolate base agar (OXOID) with bacitracin for *Haemophilus* and on the selective MacConkey agar (OXOID) for the isolation of Gram-negative bacilli. After 18–24 h of incubation in the atmosphere containing 5% CO_2_ at 37 °C the colonies were isolated. Subsequently, the bacteria were identified using a B.D. Phoenix (Becton Dickinson) automated microbiology system with appropriate test kits for Gram-negative and Gram-positive bacteria. *Haemophilus* rods were identified with discs containing factors V, X and bacitracin and on Müller–Hinton agar plates (OXOID) by incubating a McFarland 0.5 suspension with paper discs for 24 h at 37 °C with access to CO_2_. Isolation and identification of bacteria and antibiotic susceptibility testing were performed as previously described [66] according to The European Committee on Antimicrobial Susceptibility Testing (EUCAST) 6.0 [67]. Clinical breakpoints were interpreted according to EUCAST v. 8.0 [68]. For this purpose, standardized diagnostic discs containing a specific antibiotic were used. After bacterial growth was obtained, the growth inhibition zones were measured using a caliper and the results were compared to EUCAST standards. The degree of antibiotic resistance and specific resistance mechanisms were derived from the diameters of the zones of inhibition according to EUCAST.

### 4.3. Synthesis of TA-AgNPs

TA-AgNPs were obtained using TA as a reducing and stabilizing agent. In brief, 40 mL of 0.5 mM aqueous solution of TA was introduced to 320 mL of 11 mM aqueous solution of silver nitrate. The reaction mixture was dynamically stirred on a magnetic stirrer at room temperature (ca. 25 °C). Then, 30 μL of 25 wt% ammonia solution was introduced to the obtained reaction mixture. The stirring was continued for another 30 min. Finally, the obtained suspension was washed with MilliQ-water to remove unreacted reagents. The suspension was placed in an Amicon^®^filtration cell (model 8400) equipped with membranes made of regenerated cellulose with a nominal molecular weight limit of 100 kDa. The purification process was conducted under ambient conditions (25 °C). The filtration cell was placed on a magnetic stirrer and gently mixed during the purification procedure. The progress in the purification was monitored via conductivity measurements, where the conductivity was determined using a CPC-505 pH-meter/conductometer (Elmetron, Zabrze, Poland) equipped with a conductometric sensor EC-60 for every 30 mL of collected effluents. The purification process was conducted until the conductivity of the effluents stabilized at 20 μS cm^−1^ and the pH attained a value of 5.8 [5].

### 4.4. Physicochemical Characteristics of TA-AgNPs

The concentration of TA-AgNP in the stock suspension was determined with a densitometric method according to a previously established protocol [69]. Furthermore, the concentrations of Ag^+^ ions and TA-AgNPs in the suspension were validated with ICP-OES (Perkin-Elmer OPTIMA 2100DV). After the separation of TA-AgNP from the solution by ultrafiltration (30 kDa, Amicon, Merck, Darmstadt, Germany) the respective fractions were dissolved in 70% HNO_3_ and subsequently in MiliQ-water before ICP-OES analysis. From each fraction, three independent samples were collected and analyzed in triplicate. The ICP-OES analysis validated the total concentration of TA-AgNP established with the densitometric method (respectively 217 ± 3 vs. 214 mg L^−1^) and did not detect free Ag^+^ ions in the effluent within the limit of the detection method. 

The optical properties of TA-AgNP suspension were evaluated with the use of a UV-2600 spectrometer (Shimadzu, Kyoto, Japan). The morphology and size distribution of TA-AgNPs were determined based on AFM images obtained usingan NT-MDT Solver Pro Atomic Force Microscope (AFM) equipped with the SMENA SFC050L scanning head. The AFM imaging was performed in semi-contact mode using a silicon probe (polysilicon cantilevers with resonance frequencies of 240 kHz±10% or 140 kHz±10%, a typical tip curvature of 10 nm, and a cone angle less than 201). The samples for AFM imaging were prepared on PAH-modified mica sheets according to the procedure described previously [29]. The histograms were generated from the analysis of no less than 500 TA-AgNPs. The hydrodynamic diameter and zeta potential of TA-AgNPs were determined from the measurements of diffusion coefficients (*D*) and electrophoretic mobility (*μ_e_*) which were conducted using a Zetasizer Nano Z.S. instrument (Malvern Panalytical Ltd, Enigma Business Park, UK).

### 4.5. Determination of the Minimal Inhibitory Concentration (MIC) of TA-AgNPs by the Well Diffusion Method

To approximate the minimum inhibitory concentrations (MIC) of TA-AgNPs, the suspension of the tested microorganisms of 0.5 on the MacFarland optical density scale was diluted with saline 100 times to obtain a cell density of 10^6^ CFU mL^−1^ (colony forming unit) and inoculated on the surface of Müller–Hinton agar (OXOID) (Figure 4a). Next, a sterilized pipette tip was used to form wells on the agar surface with a diameter suitable for a 200 μL pipette (Figure 4b). Each well was filled with 100 μL of TA-AgNP suspensions diluted with distilled water in concentrations ranging from 5 to 100 mg L^−1^ (in a triplet for each concentration) (Figure 4c). The concentration of TA-AgNP suspensions was increased by 5 mg L^−1^ in subsequent wells. A 0.9% NaCl solution in distilled water without TA-AgNPs was used as a control sample. Next, the plates were pre-incubation for 4 h at 4 °C (Figure 4d) to provide time for soaking the TA-AgNP suspension into the agar gel and the proper diffusion of nanoparticles from the wells to the agar surface while arresting the growth of bacteria. After that time, the plates were incubated at 37 °C for 18 h (Figure 4e). The zone of inhibition of bacterial growth around the wells was measured with Skjutmatt Digital calliper (Limit). All experiments were performed in triplicate. Thus, in this paper, MIC was defined as the lowest concentration of TA-AgNPsin the well for which the zone of inhibition around the well was observed (e.g., 10 mg L^−1^ in Figure 4f). In fact, the real concentration of TA-AgNPs was inevitably diluted in the volume of the gel surrounding the well. Therefore, the value of the MIC based on the nominal concentration of the suspension introduced into the well should be treated as an upper limit of MIC. It means that the actual MIC value for each tested isolate was not higher than the lowest concentration of TA-AgNPs initially introduced into the well around which the inhibition zone was observed. This method was not applicable for *Streptococcus pneumoniae* and *Haemophilus influenzae* because of the interactions between TA-AgNPs and the growth media suitable for these species. Therefore, the results obtained for these bacteria were considered unreliable and were excluded from the analyses.

Finally, the bactericidal effect of TA alone was investigated on two reference strains (*S. aureus* ATCC 29213 and *E. coli* ATCC 25922) according to the above procedure using three distilled water solutions of TA at concentrations of 0.55 µM, 5.5 µM and 55 µM (0.94, 9.45 and 94.5 mg L^−1^). In a negative control experiment a 0.9% NaCl solution in distilled water without TA was used.

### 4.6. In Vitro Primary Cell Culture and Cell Viability Assays

Human nasal epithelial cells (HNEpC; PromoCell) were cultured in Airway Epithelial Cell Growth Medium (basal medium supplemented with BPE, EGF, insulin, hydrocortisone, epinephrine, triiodo-L-thyronine, transferrin and retinoic acid; PromoCell) at 37 °C in a 5% CO_2_ atmosphere. HNEpC cells at passage 5 were used in the experiments. The cells were plated in 48-well plates (BD Falcon) coated with type I collagen (Corning) at the initial density of 1 × 10^4^ cells/cm^2^, 24 h prior to the experiments. The growth medium was then changed to the experimental cell culture medium containing nanoparticles or vehicle control (water). The experimental medium consisted of phenol red-free DMEM, prepared from powder using water or a mixture of water and 214 mg L^−1^ TA-AgNP suspension in water and supplemented with 1 gL^−1^ glucose, NaHCO_3_ (Gibco), sodium pyruvate (Lonza), 1% bovine serum albumin (BSA), L-glutamine and P/S (50 units/mL of penicillin and 50 µg/mL of streptomycin). Water (used as vehicle control) was a component of the cell culture medium, not an additive to the medium, and as such, the osmolality of the experimental medium was not compromised. A 48 h incubation in the experimental medium had no adverse effects on the viability of the cells compared to the PromoCell culture medium. Cell culture reagents were from Sigma unless otherwise specified.

#### 4.6.1. MTT Assay

MTT assay was performed after the incubation in the presence of TA-AgNPs, as previously described [70]. Following 2 h incubation with MTT, formazan crystals were dissolved in 5 mM HCl in isopropanol and absorbance was read at 540 nm. Experiments were performed in triplicate for each experimental condition. Data represent mean values expressed as a percentage of the vehicle control ± S.D. from three independent experiments. 

#### 4.6.2. Trypan Blue Exclusion

After the 48 h incubation in the presence of TA-AgNPs, the cells were harvested with StemProAccutase (Gibco, ThermoFisher Scientific, Waltham, MA, USA), centrifuged and resuspended in DPBS. The cell suspension was diluted 1:1 with 0.4% trypan blue and counted in a hemocytometer [71].

### 4.7. Statistical Analysis

The normality of the distribution of the microbiological data was tested using the Kolmogorov–Smirnov test. The variables under consideration did not have a normal distribution; therefore, non-parametric tests were used for further analyses. The Mann–Whitney test was used for comparisons of two groups (e.g., comparisons of MIC values in Gram-positive and Gram-negative bacteria or bacteria with and without antibiotic resistance mechanisms) and the Kruskal–Wallis test was used to compare multiple groups (e.g., comparisons of MIC values between species). The tests were performed in Statistica 13.0 (TIBCO Software Inc., Palo Alto, CA, USA). A *p*-value of <0.05 was considered statistically significant. For the HNEpC viability assays, statistical analysis was performed using one-way ANOVA followed by Dunnett’s multiple comparisons test (GraphPad Prism 8.0. GraphPad Software, San Diego, CA, USA).

## 5. Conclusions

In conclusion, the results of our study showed that most pathogens isolated from CRS patients during the episodes of exacerbations were sensitive to TA-AgNPs in concentrations safe for the nasal epithelium in vitro. However, the in vitro experiments may have under- or overestimated the toxicity of antimicrobials. Therefore, further in vivo studies on the toxicity, pharmacokinetics and pharmacodynamics are required before AgNPs are approved as intranasal antimicrobials in humans.

Due to the unpredictability and significant differences in the MIC values between isolates, testing of sensitivity to AgNPs should be indicated before their application in every case. This approach may prevent exposition to suboptimal drug concentrations that promote the development of silver resistance.

## Figures and Tables

**Table 1:** The upper limit of minimal inhibitory concentration (MIC) of TA-AgNPs for the reference strains and clinical isolates (mg L^−1^). MIC values did not have a normal distribution; therefore, the median was used as a measure of central tendency and the interquartile range was used as a measure of statistical dispersion. Higher MIC values indicate a lower sensitivity to TA-AgNPs.

Species (No. of Clinical Isolates)	Reference Strain	Reference Isolates			
		Median	Min	Max	Interquartile Range
*Staphylococcus aureus* (30)	5	10	5	40	15
*Pseudomonas aeruginosa* (5)	10	5	5	20	5
*Escherichia coli* (4)	5	25	10	25	7.5
*Klebsiella pneumoniae* (3)	5	25	10	30	20
*Klebsiella oxytoca* (2)		-	10	40	-
*Acinetobacter baumannii* (2)		-	10	10	-
*Serratia marcescens* (1)		-	25	25	-
*Enterobacter cloacae* (1)		-	30	30	-
All clinical isolates (48)		10	5	40	15
All Gram-positive clinical isolates (30)		10	5	40	15
All Gram-negative clinical isolates (18)		15	5	40	15

**Table 2:** The upper limit of minimal inhibitory concentration (MIC) of TA-AgNPs for clinical isolates with and without mechanisms of antibiotic resistance (mg L^−1^). MIC values did not have a normal distribution; therefore, the median was used as a measure of central tendency and the interquartile range was used as a measure of statistical dispersion. Higher MIC values indicate lower sensitivity to TA-AgNPs. ESBL—extended-spectrum beta-lactamases; MRSA—methicillin-resistant *Staphylococcus aureus*; MLS—resistance to macrolides-lincosamides and streptogramins.

Mechanisms of Antibiotic Resistance (Number of Bacterial Isolates)	Median	Min	Max	Interquartile Range
no mechanisms of antibiotic resistance (34)	10	5	40	15
with mechanisms of antibiotic resistance (14)	17.5	5	40	15
ESBL (5)	25	25	30	2.5
MRSA + MLS (2)	-	5	25	-
MLS (7)	10	10	40	15

## Data Availability

The raw data supporting the conclusions of this article will be made available by the authors, without undue reservation; the preprint of this manuscript is available at bioRxivhttps://doi.org/10.1101/2022.01.03.474872.
